# Risk factors for migraine and tension-type headache in 11 year old children

**DOI:** 10.1186/1129-2377-15-60

**Published:** 2014-09-10

**Authors:** Karen E Waldie, John MD Thompson, Yasmine Mia, Rinki Murphy, Clare Wall, Edwin A Mitchell

**Affiliations:** 1 School of Psychology, Faculty of Science, The University of Auckland, Private Bag 92019, Auckland 1142, New Zealand; 2 Department of Paediatrics, The University of Auckland, Auckland, New Zealand; 3 Department of Medicine, The University of Auckland, Auckland, New Zealand; 4 Discipline of Nutrition, The University of Auckland, Auckland, New Zealand

**Keywords:** Migraine, Tension-type, Paediatrics, Small for gestational age, Longitudinal, Risk-factors, Paediatric

## Abstract

**Background:**

Though migraine and tension type headache are both commonly diagnosed in childhood, little is known about their determinants when diagnosed prior to puberty onset. Our aim was to determine psychosocial- and health-related risk factors of migraine and tension-type headache in 11 year old children.

**Methods:**

871 New Zealand European children were enrolled in a longitudinal study at birth and data were collected at birth, 1, 3.5, 7, and 11 years of age. Primary headache was determined at age 11 years based on the International Headache Society. Perinatal factors assessed were small for gestational age status, sex, maternal smoking during pregnancy, maternal perceived stress, and maternal school leaving age. Childhood factors assessed were sleep duration, percent body fat, television watching, parent and self-reported total problem behaviour, being bullied, and depression.

**Results:**

Prevalence of migraine and tension-type headache was 10.5% and 18.6%, respectively. Both migraine and TTH were significantly associated with self-reported problem behaviour in univariable logistic regression analyses. Additionally, migraine was associated with reduced sleep duration, and both sleep and behaviour problems remained significant after multivariable analyses. TTH was also significantly associated with antenatal maternal smoking, higher body fat, and being bullied. For TTH, problem behaviour measured at ages 3.5 and 11 years both remained significant after multivariable analysis. Being born small for gestational age was not associated with either headache group.

**Conclusions:**

Although they share some commonality, migraine and tension-type headache are separate entities in childhood with different developmental characteristics. The association between primary headache and problem behaviour requires further investigation.

## Background

Headache is one of the most frequently reported pain complaints in children and adolescents [1-3], with the prevalence increasing throughout childhood and peaking at 11–13 years of age [4]. It has been estimated that around 6.1% to 13.6% of children suffer from migraine and 9.8% to 24.7% suffer from tension-type headache (TTH) [5-8]. Despite the high prevalence, there is a paucity of data on childhood headache relative to our knowledge of adult-onset headache epidemiology. Understanding factors relating to migraine and tension-type headache prior to puberty is important because environmental factors such as stress and other family variables may not be independent of the possible effects of subsequent hormonal changes on the developing teenager [9].

Childhood headache disorders should be recognised as a significant health concern due to the considerable impact on the child and the family [10,11]. Compared to headache-free children, concerns include greater rates of absence from school [12,13], fewer and poorer peer relations including bullying [14-16], and general impairments in home life, school and leisure activities [17-20]. Although less common, migraine is considered to be more severe and disabling than TTH and involves higher rates of medication use [1].

Obesity, sleep disturbance, behavioural and psychiatric problems have all been linked with childhood migraine and/or TTH. As body mass increases, headache frequency and disability due to headache also increases [21-23]. Difficulties falling asleep and maintaining sleep, sleep breathing disorders and disorders of arousal have been particularly associated with migraine [24-26]. Behavioural problems include concentration difficulties, hyperactivity, and conduct problems [27,28]. Children with headache also have more internalizing disorders, particularly major depression [29] and anxiety [30,31], than headache-free controls. It is not clear, however, whether these associations are different between the two types of headache in children, given their potentially distinct underlying pathophysiology [32].

Toward this end, we investigated risk factors for primary headache in preadolescent children using data collected from the Auckland Birthweight Collaborative study (ABC), a longitudinal, case–control study of appropriate for gestational age (AGA) and small for gestational age (SGA) individuals born at term. SGA has been associated with numerous adverse outcomes related to cognition, health and behaviour [33-35]. As such, we studied the association of birthweight and several other antenatal, early life, and childhood risk factors with migraine and TTH at age 11 using the International Headache Society (IHS) classification system [36]. The use of a prospective longitudinal study meant that we were able to investigate factors earlier in life that may be associated with later headache diagnosis, without the problems of retrospective recall such as distortion or forgetting which may introduce bias.

## Methods

### Study cohort

The ABC study is a longitudinal prospective cohort study designed to investigate long-term growth and development in SGA and AGA children. Background information about the study design, subjects and the recruitment procedures has been described elsewhere [33]. Briefly, children born between 16 Oct.1995 and 12 Aug. 1996 to mothers who resided in the Waitemata Health or Auckland Healthcare regions were eligible for inclusion; and from 12 Aug.1996 to 30 Nov. 1997 babies born in the Auckland Healthcare region were eligible to participate. All children were born at ≥37 wk. gestation. Infants were excluded from participation if they were born from multiple births, had life threatening congenital problems, or were delivered at home. At birth, all SGA (<10% percentile for gestational age) were selected and a random sample of children born appropriate for gestational age (AGA) as controls.

Children were initially assessed at birth (Phase 1; 1995 to 1997) and during subsequent follow-ups at 12 months (Phase 2; 1996 to 1998), 3 · 5y (Phase 3; 1999 to 2001), 7y (Phase 4; 2002 to 2005) and 11y (Phase 5; 2008 to 2010). Participation rates for non-European ethnic groups were poor so analysis was restricted to European children. A total of 871 infants were born to mothers who identified themselves as European, of whom 385 (44.2%) were SGA and 486 were AGA. Of the 871 European babies enrolled at birth, 744 (85.4%) participated at 1 year, 550 (63.2%) at 3.5 years and 591 (67.9%) at 7 years of age. There were 241 (40.8%) participants at 7 years of age who were born SGA and 350 who were born AGA.

Demographic information and information about maternal health and lifestyle during pregnancy was collected by maternal interview and from obstetric records at birth. At the age of 3.5 years, the children were directly assessed on measures of cognitive and physical development. Mothers were interviewed regarding the child’s health and development and demographic information was collected. At the age of 7 and then 11 years, health, cognitive and physical abilities were again assessed directly and information was collected from a maternal interview.

Ethics approval for each phase of this study was obtained from the Northern Regional Ethics Committee. Parents and guardians were required to sign a written consent form to allow participation of their children in the study at all assessment points. Assent was obtained from each child at 11 years.

### Outcome data

Information about headache was obtained by trained interviewers conducted at the Children’s Research Centre at Starship Children’s Hospital or in the child’s home at 11 years of age. Children who indicated they suffered from headache in the past year were then asked about the nature, duration and severity of the pain based on IHS criteria [36]. Questions regarding medical history, including medical doctor diagnosis of migraine and pain medications used (prescription and over-the-counter) were also included in the headache interview.

### Covariates

Information collected at birth included socio-demographic information (e.g., school leaving age, marital status), maternal smoking (‘Did you smoke during pregnancy?” Yes/No), and maternal perceived stress during pregnancy [37].

#### Sleep duration

When participants were aged 7 and 11, average sleep duration (hours) was measured via actigraphy. Actigraphy is a non-invasive method used to study sleep-wake patterns and circadian rhythms by assessing movement. Parents completed a questionnaire on the day the actigraphy was performed, including what time the child went to bed and rose in the morning. The analysis period for the sleep data included the period from the bedtime to the rise time as given in the diary. Analysis of actigraphy data required a complete collection of data throughout the night (i.e. no time with monitor off or missing data within the scoring interval). Sleep duration was the time from sleep onset to sleep end time [38]. Sleep duration was recoded as less than 10 hours sleep or more than 10 hours average sleep at age 7 (medium split) and as less than 9.5 hours sleep or more than 9.5 hours average sleep at age 11.

#### Percent body fat

Children were assessed for percentage body fat at age 11 years using bio-electrical impedance BIM4 (Impedimed Ltd, QLD, Australia). Children were assessed according to equipment instructions, lying supine and bladder voided, with electrodes placed according to manufacturer’s instructions on the wrist and ankles. A median split of the data (Me = 25.69%, SD = 10.27) was used to create a higher and lower body fat group.

#### Television watching

Mothers of 11 year old children were asked “During a normal week, how many hours a day (24 hours) does your child watch television?” Possible responses were ‘less than 1 hour’ ‘1-3 hours’ or ‘3+ hours’.

#### Bullying

When participants were aged 11, they were asked if they had experienced physical violence, verbal teasing, sexual harassment or racist comments more than 5 times during the previous 6 months. Children who answered yes to any of these questions were classified as having been bullied.

#### Strengths and difficulties

The Strengths and Difficulties Questionnaire (SDQ) is a behavioural screening questionnaire used to measure five separate constructs of hyperactivity, emotion, conduct, peer and pro-social behaviours [39]. Questionnaires were administered at ages 3.5, 7 and 11. At ages 3.5, and 7 the questionnaire was completed by the mother of the child, while at the age of 11 separate questionnaires were completed by both the mother and the child themselves. Total difficulties were calculated by adding together the scores on the hyperactivity, emotion, conduct and peer subscales (pro social score is kept separate). SDQ scores were coded as either normal or borderline/abnormal.

#### Depression

At age 11, children in the study were classified as either depressed or not depressed by using The Centre for Epidemiological Studies Depression Scale for Children (CES-DC). It is a 20 item self-report questionnaire with scores ranging from 0 to 60, with higher scores indicating depression.

### Statistical analysis

Chi square tests for independence were conducted to assess associations between primary headache (migraine (Y/N) and TTH (Y/N)) and prenatal and lifestyle/psychosocial covariates. The factors that were significantly associated with migraine or TTH were included as predictor variables for two separate multivariable binary logistic regression analyses, each controlling for birthweight status.

Statistical significance was defined at the 5% level and analyses were carried out using IBM SPSS software, version 19.

## Results

Of the 617 participants in this study, 42.8% reported having experiencing at least one headache lasting 30 minutes or more in the past year (n = 264). From these, 10.5% were classified as experiencing migraines (n = 65) and 18.6% were classified with TTH (n = 115).

Table 1 presents descriptive statistics for sex, pain duration, and parental socio-demographic information. Males were slightly more likely to experience both migraine (n = 34, 52.3%) and TTH (n = 67, 58.3%) than females, but this difference was not statistically significant. Males reported significantly greater pain intensity during headache than females (t_(266)_ = 2.17, p = .03) but there were no sex differences in headache duration. As expected, those categorised as having migraine were significantly more likely to have been given a medical diagnosis of migraine in the past than those with TTH. Children with migraine were also significantly more likely to complain of stomach aches than those with TTH. There were no differences between groups in family socioeconomic status, maternal school leaving age or marital status.

**Table 1:** Descriptive statistics (percent in parentheses) for pain duration and socio-demographic variables as a function of primary headache

	**Migraine**	**Tension type**	
	**N = 65 (10.5%)**	**N = 115 (18.6%)**	p-value*
Duration (30 mins – 1 hr)	6 (9.2)	62 (52.5)	<.001
Duration (1 – 72 hrs)	59 (90.8)	56 (47.5)	<.001
Given over-the-counter medication	57 (87.7)	102 (85.7)	>.05
Given medication prescribed by a medical doctor	7 (10.8)	14 (11.7)	>.05
Diagnosed with migraine by a medical doctor	10 (15.4)	5 (4.2)	.01
Frequent stomach aches in past year	35 (53.8)	38 (31.9)	.005
Marital status of parents: married/de factor	62 (95.4)	111 (92.5)	>.05
Marital status of parents: single and other	3 (4.6)	9 (7.5)	>.05
Mothers first child	39 (60.0)	67 (55.8)	>.05
Maternal school leaving age			
Younger than 16	8 (12.3)	15 (13.2)	>.05
Older than 16	57 (87.7)	99 (86.8)	

*from univariable Chi Square analyses.

### Univariate analyses

Results of Chi Square univariable analyses are presented in Table 2. There were no differences in headache classification (migraine versus headache-free and TTH versus headache-free) for those participants who were born SGA compared with those who were born AGA. Maternal perceived stress (measured at birth, ages 3.5 and 7 years) were not associated with migraine or TTH.

**Table 2:** Headache classification at age 11 years in univariable analyses

	**No headache**	**Migraine**	**χ**^**2**^	**OR**	**TTH**	**χ**^**2**^	**OR**
Maternal smoking during pregnancy							
			0.67			7.05**	
No	279 (80.4%)	53 (81.5%)		1.89	78 (68.4%)		0.93
Yes	68 (19.6%)	12 (18.5%)		ref	36 (31.6%)		
Maternal perceived stress during pregnancy^†^			2.08			3.33	
Low (scores 0–13)	195(55.9%)	34 (60.9%)		ref	61 (65%)		ref
High (scores 14–39)	154 (44.1%)	29 (39.1%)		1.49	52 (54%)		1.49
Birth weight			0.11			1.22	
AGA	214 (60.6%)	38 (58.5%)		ref	63 (54.8%)		ref
SGA	139 (39.4%)	27 (41.5%)		1.09	52 (45.2%)		1.27
Gender			0.16			2.62	
Female	178 (50.4%)	31 (47.7%)		ref	48 (41.7%)		ref
Male	175 (49.6%)	34 (52.3%)		1.12	67 (58.3%)		1.42
SDQ Total difficulties at 3.5 years, parent^a^			0.63			4.60*	
Normal	239 (83.6%)	44 (88.0%)		ref	63 (73.3%)		ref
Borderline/Abnormal	47 (16.4%)	6 (12.0%)		0.69	23 (26.7%)		1.86
Maternal perceived stress at 3.5 years^†^			0.39			1.17	
Low (scores 0–13)	167 (48.1%)	33 (52.4%)		ref	61 (54%)		ref
High (scores 14–39)	180 (51.9%)	30 (47.6%)		1.08	52 (46%)		1.24
SDQ Total difficulties at 7 years, parent^a^			0.52			0.19	
Normal	274 (87%)	45 (83.3%)		ref	86 (88.7%)		ref
Borderline/Abnormal	41 (13%)	9 (16.7%)		1.34	11 (11.3%)		0.86
Sleep duration at age 7^‡^			3.82*			0.25	
<10 hours	121 (43.1%)	29 (58.0%)		1.83	41 (46.1%)		1.13
=/>10 hours	160 (56.9%)	21 (42.0%)		ref	48 (53.9%)		ref
Maternal perceived stress at 7 years^†^			0.11			0.86	
Low (scores 0–13)	69 (48.3%)	17 (51.5%)		ref	29 (55.8%)		ref
High (scores 14–39)	74 (51.7%)	16 (48.5%)		0.40	23 (44.2%)		0.81
Sleep duration at age 11^‡^			0.06			0.01	
<9.5 hours	68 (46.6%)	11 (44%)		0.90	28 (47.5)		1.04
=/>9.5 hours	78 (53.4%)	14 (56%)		ref	31 (52.5)		ref
Depression at 11 years			3.57			0.02	
No depression	317 (94.1%)	56 (87.5%)		ref	105 (93.8%)		ref
Depression	20 (5.9%)	8 (12.5%)		2.26	7 (6.3%)		1.06
Bullied at 11 years			0.81			7.73**	
No	222 (68.1%)	36 (62.1%)		ref	57 (53.3%)		ref
Yes	104 (31.9%)	22 (37.9%)		1.30	50 (46.7%)		1.87
Television watching at 11 years			0.62			0.96	
<1 hour	86 (27.3%)	13 (24.1%)		ref	22 (22.4%)		ref
1-3 hours	198 (62.9%)	34 (63.0%)		1.14	65 (66.3%)		1.28
3-5 hours	31 (9.8%)	7 (13.0%)		1.49	11 (11.2%)		1.39
Percent body fat at 11 years			2.38			9.62*	
Lower than median	180 (54.2%)	27 (43.5%)		ref	40 (37.0%)		ref
Higher than median	152 (45.8%)	35 (56.5%)		1.54	68 (63.0%)		2.01
SDQ Total difficulties at 11 years, self^a^			24.68**			4.27*	
Normal	298 (84.9%)	38 (58.5%)		ref	88 (76.5%)		ref
Borderline/Abnormal	53 (15.1%)	27 (41.5%)		4.0	27 (23.5%)		1.73
SDQ Total difficulties at 11 years, parent^a^			1.15			0.09	
Normal	306 (87.9%)	54 (15.9%)		ref	99 (86.8%)		ref
Borderline/Abnormal	42 (12.1%)	11 (23.1%)		1.48	15 (13.2%)		1.10

*Note*. *Significant at *p* < .05; ** *p* < .01; *χ*^*2*^ *=* Pearson chi square.

^†^Perceived stress in mothers categorised according to a median split.

^‡^Sleep duration categorised according to a median split and rounded to the nearest hour.

^a^Scored using the Strengths and Difficulties Questionnaire (SDQ).

Maternal smoking during pregnancy was significantly associated with TTH (p = .008), with 31.6% of TTH sufferers having a mother who smoked during pregnancy compared to 19.6% of children with no headache. Maternal smoking in pregnancy was not associated with migraine.

Sleep duration at age 7 was marginally associated with migraine (p = .05) but not TTH. Of those with migraine, 58.0% slept for less than 10 hours per night compared to 43.1% of those with no headache disorder. Sleep duration at age 11 was not associated with migraine or TTH. Average hours of television watching per day at age 11 were not associated with migraine or TTH.

The parent-scored SDQ total difficulties score at 3.5 years was significantly associated with TTH (p = .032) but not migraine. Of those with TTH, 26.7% had a borderline/abnormal score compared to 16.4% with no headache. The parent-scored SDQ total difficulties score at 7 years was not associated with primary headache.

When children themselves completed the SDQ at 11 years, the total difficulties score was significantly associated with both TTH (p = .039) and migraine (p < .001). For those with migraine, 41.5% had a borderline/abnormal score. For those with TTH, 23.5% had a borderline or abnormal score compared with 15.1% of those with no headache disorder. The hyperactivity subscale was the only subscale individually related to any headache group, with children rating themselves with borderline or abnormal hyperactivity symptoms 2.29 times more likely to have a migraine diagnosis than those without headache (p = .007).

Being bullied in the past 6 months at age 11 was significantly related to TTH (p = .005) only, with 46.7% of children with TTH experienced bullying compared to 31.9% of children with no headache.

Symptoms of depression at age 11 were slightly but not significantly related to migraine (p = .06) only, with 12.5% of children with migraine were classified as depressed compared to 5.9% of children with no headache.

Percentage body fat measured at age 11 was significantly associated with TTH but not with migraine. Children with TTH had greater percentage body fat (mean = 29.28%, standard deviation (SD) =11.49) than children without headache (mean = 26.21%, SD = 9.84; t(463) = 2.70, p = .007). Interestingly, there was no relationship between body fat and IQ measured at age 11 (WISC-R) for those with TTH (r = -.102), but there was for children with migraine (r = -.303, p = .015).

### Multivariate analyses

The variables significantly associated with migraine from the univariable analyses were sleep duration age 7 and self-completed SDQ total difficulties score at age 11.

The results of the multivariable logistic regression analysis for migraine are presented in Table 3 and both variables remained significant. Children with less than 10 hours of sleep at night when they were 7 years of age were more than 2 times more likely to complain of migraine headache than children who slept longer. A borderline/abnormal total difficulties score was associated with an almost 4 times increased likelihood of suffering from migraine compared with those who had a total difficulties score in the normal range. When the hyperactivity subscale was entered separately into the model, both sleep and hyperactivity (OR 2.70 95% CI 1.28 – 5.70) remained significant.

**Table 3:** Multivariable logistic regression results for migraine headache

					**95%**	
	***B***	***SE***	**Wald statistic**	**Odds ratio**	**Confidence interval**	
SGA/AGA	0.11	0.33	0.11	1.12	0.59	2.13
Sleep duration (at 7 years)	0.73	0.32	5.09	2.08*	1.10	3.94
Total difficulties (at 11 years) self-completed	1.48	0.34	19.27	4.41*	2.27	8.54

*p < .05.

The variables significantly associated with TTH in univariable analyses were maternal smoking during pregnancy, total difficulties at 3.5, child-scored total difficulties score at 11, percent body fat at 11, and bullying at 11. The results of the multivariable logistic regression analysis for TTH are presented in Table 4. Only total difficulties at 3.5 and 11 remained significant predictors of TTH. Children who had borderline/abnormal total difficulties score at age 3.5 were 4 times more likely to suffer from TTH compared with those in the normal range. This trend was consistent when children themselves rated their difficulties at age 11. Children in the borderline/abnormal range were over 4 times more likely to be classified with TTH than children in the normal difficulties range.

**Table 4:** Multivariable logistic regression results for tension type headache

	***B***	***SE***	**Wald statistic**	**Odds ratio**	**95%**	
					**confidence interval**	
Birthweight	-0.18	0.47	0.14	1.11	0.58	2.12
Smoking during pregnancy	0.46	0.61	0.58	1.59	0.48	5.21
Total difficulties (at 3.5 years)	1.38	0.62	5.02	3.96*	1.19	13.21
Percent body fat (at 11 years)	0.34	0.43	0.63	1.41	0.76	4.72
Bullying (at 11 years)	0.64	0.46	1.91	1.90	0.60	3.28
Self-reported total difficulties (at 11 years)	1.48	0.34	19.27	4.41*	1.10	8.54

*p < .05.

## Discussion

Migraine is an episodic and disabling neurological condition affecting about 14% of the adult population [40] while TTH is even more prevalent than migraine [41]. Relative to adults, little is known about the epidemiology of primary headache in children. We found that migraine was associated with both reduced sleep duration and problem behaviour. TTH was associated with antenatal maternal smoking, problem behaviour, higher body fat, and being bullied.

Just under half (48%) of 11 year old ABC study members had reported experiencing at least one headache lasting half an hour or more in the past year. From this, the prevalence of primary headache was 10.5% for migraine and 18.6% for TTH. These rates accord well with other studies [1-8]. Males and females were almost equally likely to report symptoms of migraine and TTH. Our lack of sex difference is consistent with previous research [1,4,31]. Headache prevalence in females tends to increase with age [42]. After about 12 years of age the female to male ratio is 1:3 for migraine and 4:5 for TTH [41-43]. This is most likely due to hormonal changes during puberty [44,45] but may also be related to gender differences in cognitive and social reactions to pain [46].

Family socio-economic status was not significantly associated with either migraine or TTH, possibly due to the small sample sizes in the lower socio-economic group or because the components of SES likely to be contributing to headache is more accurately captured by other factors. This finding is consistent, however, with a number of studies that failed to establish a link between socioeconomic status and headache [2,17,28,31]. A population based study of Finnish school children did find a higher risk of TTH for those children whose fathers were lower level white collar workers as opposed to independent traders/employees, but found no association between episodic TTH and family unemployment, economic problems and single parent families [7]. We similarly found no association between headache and marital status.

Little is known about the influence of prenatal events on the development of migraine and TTH in children. Though studies have found evidence of a link between maternal stress during pregnancy and negative outcomes in children [47], we did not find any association between perceived stress during pregnancy and offspring headache. There was also no difference in the rates of primary headache between the SGA and AGA groups.

Maternal smoking during pregnancy was significantly associated with TTH but not with migraine. Arruda and colleagues [48] similarly found that exposure to prenatal tobacco was a risk factor for chronic daily headache in children. It is unclear whether this association is due to socioeconomic factors or is specifically health-related. Women who continue smoking throughout pregnancy are generally of lower age, socio-economic status, level of education and occupational status than non-smokers [49], and offspring are exposed to higher levels of social disadvantage and family dysfunction throughout childhood [50].

It may also be that smoking during pregnancy has affected the child’s neonatal environment, predisposing them to develop headaches later in life. Smoking during pregnancy has been linked to increased risk of numerous childhood problems including asthma [51], attention deficit disorder [52] and conduct disorder [50].

Percentage body fat at age 11 was associated with TTH. These findings are supported by research by Hershey and colleagues [53], who found that body mass was positively associated with headache frequency in 913 children recruited from paediatric headache centres. Furthermore for those children who were defined as overweight in the first visit, drops in weight were significantly associated with decreases in headache frequency at both 3 and 6 months follow up. Earlier studies have also found associations between obesity and migraine frequency among children [21-23] but there are no known reports of increased frequency or prevalence of TTH among overweight/obese children or adults. It may be that children who have more body fat may also have higher stress due to a lower self-esteem. It should be noted that this variable didn’t remain significant in our multivariable analysis, possibly due to low statistical power. Further research with larger samples is needed to determine the relationship between body weight and headache disorder and the mechanism behind it.

One possible consequence of being a child with higher body fat might be increased susceptibility to bullying by peers. We found that bullying was not associated with migraine but was significantly associated with TTH. Children with TTH tend to be shier and less sociable than children with migraine [28]. Further research is needed to determine whether severity of bullying is associated with headache prevalence or frequency and whether bullying is consistently associated with TTH rather than migraine.

Children with migraine were more likely to sleep for less than 10 hours each night when they were 7 years of age compared to those with no headache disorder. Several prior studies have also demonstrated the co-occurrence of sleep disturbances and migraine in children [2,24]. Although children with migraine in our study slept less than children without headache, this did not seem to be related to increased time watching television. Others have similarly found no relationship between migraine or TTH and hours spent using electronic equipment such as television/computer/PlayStation [17]. One possible reason that our migraineurs are getting less sleep is that they may also suffer from a periodic limb movement disorder [54]. This possibility will be tested at the next ABC medical assessment.

The co-morbidity of sleep disorders and migraine in childhood has led to the suggestion that they may have a common genetic or pathophysiological mechanism [26]. The serotonergic system is likely to be involved, as it has been implicated in the physiology of migraine and plays an important role in the initiation and maintenance of sleep activity [26]. Serotonin also plays an important role in emotional disorders such as depression, which has also been implicated in the physiology of migraine [31] and studies with adults have found that depression precedes migraine onset [55]. Although we did not find depression was related to childhood migraine or TTH, analyses with larger samples are needed to confirm this and to determine whether serotonin links the underlying pathophysiology between migraine, sleep problems and emotional disorders.

Though we didn’t find an association between headache and depression, our results indicate that many other areas of psychological functioning were significantly associated with childhood headache. This is consistent with a growing body of literature suggesting a link between childhood headache disorders and emotional and behavioural difficulties [27-31]. In our study, psychological functioning was assessed by parent report at ages 3.5, 7, and 11 (and self-report at 11) using the SDQ. This questionnaire assesses emotional symptoms, conduct problems, hyperactivity/impulsivity, and peer problems. Scores from these subgroups are summed to provide a total difficulty score.

More specifically, migraine and TTH were both significantly associated with self-reported borderline/abnormal score even after controlling for other risk factors. An earlier clinical study of behavioural and emotional difficulties found that children with TTH have more behavioural, emotional and temperamental difficulties than those with migraine [28] while a later study from a paediatric headache centre found the reverse [31]. In particular, migraine was associated with a borderline/abnormal score on the hyperactivity/impulsivity subscale of the SDQ, consistent with an earlier study [56]. Despite some inconsistencies, findings suggest that primary headache in childhood is complicated by, or occurs due to, a combination of a predisposition and psychosocial and behavioural difficulties.

## Conclusions

Our findings support the view that migraine and TTH should be considered separate entities in childhood [but see 17 for an alternate view]. While both migraine and TTH were associated with more self-reported total difficulties at age 11 years, we found a number of independent risk factors. TTH was associated with smoking during pregnancy, parent-reported total difficulties at age 3.5 years, higher percent body fat, and being bullied at age 11. Migraine was independently associated with reduced sleep duration at 7 years, consistent with reports that psychological problems and sleep issues are often linked with migraine.

A number of important limitations of our study need to be discussed. Firstly, although the overall sample size was large (n = 617), the combination of the prevalence of headache type and other factors with low prevalence such as depression resulted in small case sizes for those factors. Secondly, our participants were from New Zealand European families and did not include Maori and Pacific families. This was due to low response rate from non-European families during the earlier follow-up phases. Thirdly, we did not have access to medical information from first degree relatives and therefore could not include family history of headache in our analyses. Fourthly, our findings are correlational and should not be interpreted as factors that necessarily cause headache disorder.

Despite the many limitations, there were some particular strengths of our study. For example, much of the research into childhood headaches is conducted using clinical samples from speciality headache centres. Results from these studies may therefore not generalize to the general population. Moreover, many studies do not differentiate between general headache and disorder subtypes, or they use non-standardised classification systems to diagnose headache, making it difficult to compare results across studies. Broadly representative cohort studies using internationally recognized headache diagnostic criteria might more reliably determine if differences exist between children who suffer from primary headaches and from those who do not and whether differences exist between headache groups.

In summary, a number of health-related and psychosocial risk factors were associated with childhood headache in our cohort study. Future studies are planned to determine if there is an association between primary headache and common illnesses/diseases in childhood. Further work to determine risk factors of migraine and TTH will help ensure that earlier diagnosis and effective treatment occurs.

## Abbreviations

ABC: Auckland Birthweight Collaborative; AGA: Appropriate for gestational age; IHS: International Headache Society; SGA: Small for gestational age; SDQ: Strengths & Difficulties Questionnaire; TTH: Tension-type headache.

## Competing interests

The authors declare that they have no competing interests.

## Authors’ contribution

KW wrote the first draft of the manuscript and supervised the student working on a subset of this data. JMD is the Deputy Director of the ABC study and oversaw all data analyses. YM is the Masters student who conducted analyses for a subset of the analyses in this manuscript and contributed to some of the writing. CW contributed to the design of the study and edited a draft of the manuscript. RM contributed to the design of the study and edited a draft of the manuscript. EAM is the Director of the ABC study, contributed to the design of this study, and read and approved the final manuscript. All authors read and approved the final manuscript.
